# Social and cultural barriers to husbands’ involvement in maternal health in rural Gambia

**DOI:** 10.11604/pamj.2017.27.255.11378

**Published:** 2017-08-07

**Authors:** Mat Lowe

**Affiliations:** 1 Society for the Study of Women’s Health (SSWH), Old Yundum, The Gambia

**Keywords:** Maternal health, The Gambia, husbands, male involvement, qualitative research

## Abstract

**Introduction:**

While many studies have documented a number of socio-cultural barriers to male involvement in maternal health, in The Gambia very little information is known about the social and cultural practices that characterized male involvement in maternal health. This study aims to explore some of the underlying social and cultural factors affecting husbands’ involvement in maternal health issues pertaining to pregnancy and delivery in rural Gambia.

**Methods:**

Five focus group discussions and six in-depth interviews were conducted among rural men and traditional birth attendants in five areas of rural Gambia. The discussion was directed to the roles of male partners in pregnancy and delivery and the difficulties they face regarding taking care of their wives. The data resulting from the discussion was audio-recorded, transcribed verbatim, and analyzed thematically.

**Results:**

In general, rural Gambian men and traditional birth attendants (TBAs) reported that husbands’ involvement in maternal health is highly desirable, but is influenced by many factors, such as the traditional conceptualization associated with pregnancy and delivery as women’s domain. In addition, many men do not believe that pregnancy chores warrant their efforts compared to other competing social responsibilities. This issue may be more complicated in polygamous marriages where there is rivalry among co-wives and in neighborhoods where men who help with house chores may be subjected to mockery.

**Conclusion:**

These findings suggest that husbands’ involvement in maternal health in The Gambia is influenced by the prevailing social and cultural practices of gender role and norms, which are also at the root of maternal health problems.

## Introduction

The relationship that male partners have to women’s health and health seeking behavior is important to examine, both for the way that it reflects the practices of gender inequalities within the family and the community, and for the way that it affects women’s health [1]. This study relates to the International Conference on Population and Development (ICPD), which was held in Cairo, Egypt in 1994. The ICPD Program of Action recognizes the importance of men to women’s reproductive health and draws attention to the unfairness inherent in many men’s and women’s gender roles. Since then, various policy and programmatic interventions that aimed at creating a space and engaging men in maternal health have been adopted in various countries [2].

In The Gambia, for instance, the National Reproductive Health Policy (2007/2014), was aimed among other things at addressing the low level of male participation in reproductive and child health services. However despite this significant stride, there is minimal support from men in women’s experiences of pregnancy, childbirth, and the postnatal period in The Gambia [3]. Many studies conducted in other countries have shown a variety of factors that hinder men from participating in pregnancy and childbirth [4]. These include lack of space in some health facilities to accommodate male partners, or the unfriendly attitude of some healthcare provider [5, 6]. Other proximal determinants include the traditional perceptions associated with pregnancy and delivery, type of marital practice, age compatibility between husband and wife, men’s conflicting work obligation and low knowledge levels of pregnancy, and social stigma [7, 8]. Although many of these studies have identified a number of health service and socio-cultural barriers to male involvement in pregnancy and delivery; studies conducted in The Gambia have almost entirely focused on the barriers in health services [9, 10], with little attention to the social and cultural practices. In rural areas. In particular, there is relative scarcity of information regarding men’s views on the roles of male partners in delivery in The Gambia [3]. This study sought to address this gap in the literature by exploring, through the perspectives of rural men and traditional birth attendants, the underlying social and cultural factors affecting husbands’ involvement in maternal health issues pertaining to pregnancy and delivery in rural Gambia.

## Methods

The study was part of a larger exploratory qualitative study on the social and cultural factors affecting maternal health in rural Gambia [11]. The Gambia, like many other countries in sub-Saharan Africa has long been overburdened with maternal health issues [11]. The national mortality ratio, which has fallen by 46% over the last two decades, is among the highest in Africa [9]. The major causes of maternal mortality in The Gambia include restricted access to emergency obstetric care [9], substandard quality of referral care [12], hemorrhage and related conditions such as hypertension and anemia [13], malaria during pregnancy and hepatitis [14]. This study was conducted in five areas of rural Gambia (Makka Farafenni, Kerr Ardo, Kerr Gumbo, Mballow Omar and Bakindik) in the North Bank Region. These five rural communities were chosen based on their long-standing history of research intervention and continuous demographic surveillance [15]. It is also common to find women serving non-governmental organizations in these rural communities for research collaboration. The study partnered with the Agency for the Development of Women and Children (ADWAC) in order to have easy access to the study communities and participants.

The participants comprised of fifty (50) married men who were recruited by ADWAC field coordinators. They were all agricultural and rural by residence, but varied in number of wives and educational level. Only one out of the fifty participants has high school education and the rest have no or few years of primary or informal (Arabic) education. They were purposively recruited based on their marital status as husbands and household heads. It was assumed that these characteristics would provide them with knowledge and experience of the roles of male partners in pregnancy and delivery. Six traditional birth attendants (TBAs) were also recruited. The TBAs were included in this study based on their experience in carrying out home deliveries as well as their social cohesion role in rural Gambian societies [16].

The study was approved by The Gambia Government/Medical Research Council Joint Ethics Committee. Verbal informal consent was sought and obtained for individual participation prior to each focus group discussion and in-depth interview, with confidentiality of the data ensured all throughout. The data collection was conducted during August and September 2012, in partnership with the ADWAC. Five focus group discussions (FGDs) were conducted among fifty married men. The focus group discussions were held in the morning before the men started to work in the field and sometimes in the afternoon, and lasted typically for 70-90 minutes, although in one occasion it was only 45 minutes. Each focus group discussion was limited to ten participants for ease of management. Following informed consent routines, participants were asked to talk about their perception of the role of male partners in pregnancy and delivery and the difficulties they face taking care of their wives during pregnancy and delivery. This discussion was supplemented by interactive questions about such topics as attending prenatal care and deliveries and providing escort to health facilities.

In-depth interviews (IDIs) were also conducted for six traditional birth attendants (TBAs) for in-depth information on the topic of male involvement in maternal healthcare and the challenges of involving men in maternal healthcare. The in-depth interviews with TBAs were conducted to enrich data from the FGDs and to facilitate data triangulation [17]. The data collection process for both the FGDs and IDIs was facilitated by an audio-recorder with two research assistants; a female nurse and a male community development worker. The research assistants had mastery of the two local languages (Wolof and Mandinka), with experience in data collection. For the data analysis, all sound recording files from the focus group discussions (FGDs) and in-depth interviews (IDIs) were transcribed verbatim and translated from vernacular language into English. Qualitative thematic data analysis was done for both FGDs and IDIs, in which common properties that bear similar ideas were grouped into key concepts. Concepts with common properties were then grouped into the main themes. Participants quotes were reported directly as were spoken without editing the grammar, to avoid losing meaning [4].

## Results

A good part of participants’ description of the social and cultural factors affecting husbands’ involvement in maternal health include: (a) the general perception associated with pregnancy and delivery as women’s domain, (b) husbands’ competing job responsibilities, (c) rivalry among co-wives, and (d) fear of mockery.

### Pregnancy and delivery as women’s domain

While the participants have demonstrated desirability to get involved in issues of pregnancy and delivery, the general conceptualization associated with pregnancy and delivery posed a significant obstacle to their full involvement. The participants in several focus groups reported that when it comes to antenatal visits they were usually involved in providing transportation or monetary fee. However, hardly were they involved in spontaneous normal or complicated deliveries involving prolonged labor or bleeding. In either case, pregnant women were usually escorted to health facilities by traditional birth attendants. One of the participants vividly explained this situation in the following: “*I sometimes take my wife to the clinic for antenatal check-ups, but to be honest when it comes to the delivery it is usually the traditional birth attendant who takes her to the clinic*”. [FGD, 1]

Another participant added: “*I think that pregnant women should be taken to the health facility by their fellow women especially during delivery. It is better to leave it that way since pregnancy and delivery is not our (husbands’) responsibility*”. [FGD, 1]

This kind of testimony is not uncommon and is culturally motivated. A TBA in one of the interviews recounted a scenario in which she insisted on a husband to attend the delivery of his child but the husband insisted on not attending for the simple reason that he is culturally forbidden to enter the delivery room. The TBA narrated: “*I insisted on him several times, but he said he cannot enter the delivery room, or witness the birth of his child. I think there are some mystical reasons associated with this* [IDI, 3]”. Numerous testimonies like these suggested that gender role norms appeared to post a significant barrier towards husbands’ involvement in delivery, but other reasons may have also contributed to their limited involvement.

### Husbands’ competing job responsibilities

Though many reasons why it was important to escort their wives to health facilities for antenatal exams and delivery purposes were put forward by participants, the majority reported that they were usually constrained by their work obligation. Many participants reported that they have not taken up pregnancy chores simply because they do not see the need or believe that pregnancy chores warrant their efforts against other competing job responsibilities. One of the participants, a father of four kids explained in the affirmative: “*To be honest, I have never witnessed the birth of my child; it always meets me at my work place*”. [FGD, 5]

As a result, women issues were placed as less preoccupying for men and they tended to follow without question the traditional male-female social expectation of gender roles. One of the participants, a polygamous man with five kids vividly stated this issue in the following: “*It is not that we do not want to get involved, but because we have other things to do”*. [FGD, 4].

For this reason, most men were not aware of their wives’ pregnancy until it reaches a rather late stage. One of the TBAs explained: “*I once escorted a woman to the health facility, but until the time she delivered and even after that the husband did not come. When I called him; he said he did not know that his wife was approximating her delivery date. He was busy working on the farms at the time*”. [IDI, 6].

This and other related testimonies showed remoteness and limited gender interaction between husband and wife regarding pregnancy issues. It also suggests that as heads of households, men were limited in their resources including income, time and opportunities to attend deliveries. These issues may be more complicated in polygamous marriages where there is rivalry among co-wives.

### Rivalry among co-wives

In many focus groups, the rivalry among co-wives in polygamous marriages was among the first issues to be brought up by the participants. Polygamous men in several group discussions reported that their involvement in pregnancy and delivery are limited by the unhealthy competition among co-wives in polygamous households. Several participants raised concerns about the difficulties involved in satisfying women in their needs and this issue becomes more complicated with two wives. One of the participants, a polygamous man explained: “*Women are just like kids; very difficult to satisfy in their needs. For example, if you escort one of your wives to the health facility for medical check-up, or provide her with money for antenatal exams, the other co-wife will also expect the same. And if you cannot do the same for the second wife it becomes a serious issue” (FGD, 2)*.

As a result, polygamous men could not fully be involved in issues of pregnancy and delivery so as to maintain stability in their marriages. This was also another reason why household duties for pregnant women were not alleviated by either their husbands or the other members of polygamous households. The TBAs in several in-depth interviews reported that pregnant women did not enjoy privileges in the household when they were pregnant due to limited assistance they received from their husbands or other household members. This limited assistance may mean that pregnant women need to work exceedingly hard, with occasions of pregnancy and birth related complications. For instance, the physicality of women’s work burden was explained by this TBA who echoed: “*You can be in your ninth month of pregnancy but still you have to pound grains like coos or maize and go to the farm every day. Sometimes we send women to the hospital and at the hospital it is found out that they either have no water (dehydrated) or insufficient blood (anemic)*. [IDI, 5].

Numerous testimonies like these indicated some of the difficulties encountered by pregnant women regarding workload and the lack of flexibility in job arrangement within the household, which could be because men did not have full understanding of women’s matter or because they are afraid of been mocked at when they participate in house chores.

### Fear of mockery

According to our interviews with TBAs, men helping with household chores may be subjected to mockery. One of the TBAs expounded: “Our society is just too complex. Even if some men want to assist their wives with domestic chores relating to pregnancy it is the people, sometimes their fellow men who will discourage them from doing so”. (IDI, 3). Another man with few years of high school education added: “*Sometimes you may want to help your wife or even take her to the clinic for antenatal exams, but if you come to think of what other people would say that alone can prevent you from doing it*”. (FGD, 2). In view of the above findings, antenatal or delivery attendance by husbands was limited in these rural communities, and taken together these issues highlighted some of the underlying social and cultural factors affecting husbands’ involvement in maternal health in The Gambia, which ultimately has implications for maternal morbidity and mortality.

## Discussion

The aim of this study was to explore some of the underlying social and cultural factors affecting husbands’ involvement in maternal health issues pertaining to pregnancy and delivery in rural Gambia. The findings revealed that husbands’ involvement in maternal health is desirable, but is affected by a confluence of factors [1]. In particular, the traditional conceptualization associated with pregnancy and delivery [18], men’s reluctant to acknowledge either that they have a role to play in pregnancy and delivery were among the most salient points. The study also found that as household heads, husbands were limited in their material and social resources including income, time and opportunities to attend deliveries or escort their wives to health facilities for antenatal exams. These same factors were responsible for the limited gender interaction between husband and wife regarding pregnancy issues. The majority of participants reported that they are usually not aware of their wives’ pregnancy until it reaches a rather late stage, indicating remoteness and lack of communication between husband and wife. In addition, husbands’ roles are also shaped and restricted by cultural practices: according to our interviews, men who help with household chores may be subjected to mockery in the neighborhood. Social stigma against men who engage in household chores is a prominent social barrier that inhibits male participation in maternal health [7, 19]. Male involvement in maternal health is also complicated by the rivalry between co-wives [20], which is one reason why household duties for pregnant women were not alleviated by either their husbands or the other members of polygamous households [11]. Polygamous women in this regard are also less likely to obtain money from their husbands for treatments requiring monetary fee [21]. This may mean that women need to work daily in order to save money for their medical and other related expenses, with occasions of pregnancy and birth-related complications [11].

## Conclusion

Although the study does not have concrete data, it is believed that the participants reflected faithfully on some of the social and cultural factors affecting husbands’ involvement in maternal health. The study, however, is limited in its research design which must be considered. First, it was conducted in a predominantly male dominated society. The limitation of applying the findings in a more pragmatic and egalitarian society is therefore acknowledged [6]. Second, the findings are based on individuals’ reports and not on close observation of what actually happens in rural households [21]. Nonetheless, the results hold important implications for policy and or practice.

### What is known about this topic

- Husbands’ involvement in maternal health is influenced by a number of health service and socio-cultural factors.

### What this study adds

- That, in The Gambia, husbands’ involvement in maternal health issues is highly desirable. However, it is affected by the prevailing socio-cultural practices of gender role and norms, which are also at the root causes of maternal health problems.

## Competing interests

The author declares no competing interest.
